# Anti-integrin αvβ6 autoantibodies are a potential biomarker for ulcerative colitis-like immune checkpoint inhibitor-induced colitis

**DOI:** 10.1038/s41416-024-02647-1

**Published:** 2024-03-09

**Authors:** Masataka Yokode, Masahiro Shiokawa, Hisato Kawakami, Takeshi Kuwada, Yoshihiro Nishikawa, Yuya Muramoto, Hiroki Kitamoto, Makoto Okabe, Hajime Yamazaki, Norihiro Okamoto, Toshihiro Morita, Kazuya Ohno, Risa Nakanishi, Ikuhisa Takimoto, Muneji Yasuda, Koki Chikugo, Shimpei Matsumoto, Hiroyuki Yoshida, Sakiko Ota, Takeharu Nakamura, Hirokazu Okada, Tomonori Hirano, Nobuyuki Kakiuchi, Tomoaki Matsumori, Shuji Yamamoto, Norimitsu Uza, Makoto Ooi, Yuzo Kodama, Tsutomu Chiba, Hidetoshi Hayashi, Hiroshi Seno

**Affiliations:** 1 Department of Gastroenterology and Hepatology, Kyoto University Graduate School of Medicine, Kyoto, Japan; 2 Department of Medical Oncology, Kindai University Faculty of Medicine, Osaka, Japan; 3 Section of Clinical Epidemiology, Department of Community Medicine, Graduate School of Medicine, Kyoto University, Kyoto, Japan; 4 Division of Gastroenterology, Department of Internal Medicine, Kobe University Graduate School of Medicine, Hyogo, Japan; 5 Department of Gastroenterology and Hepatology, Kitano Hospital, Tazuke Kofukai Medical Research Institute, Osaka, Japan; 6 Department of Gastroenterology, Shizuoka General Hospital, Shizuoka, Japan; 7 Department of Gastroenterology and Hepatology, Kansai Electric Power Hospital, Osaka, Japan

**Keywords:** Diagnostic markers, Autoimmunity

## Abstract

**Background:**

No specific biomarker for immune checkpoint inhibitor (ICI)-induced colitis has been established. Previously, we identified anti-integrin αvβ6 autoantibodies in >90% of patients with ulcerative colitis (UC). Given that a subset of ICI-induced colitis is similar to UC, we aimed to clarify the relationship between such autoantibodies and ICI-induced colitis.

**Methods:**

Serum anti-integrin αvβ6 autoantibody levels were compared between 26 patients with ICI-induced colitis and 157 controls. Endoscopic images of ICI-induced colitis were centrally reviewed. Characteristics of anti-integrin αvβ6 autoantibodies in the ICI-induced colitis patients were compared with those of UC patients.

**Results:**

Anti-integrin αvβ6 autoantibodies were found in 8/26 (30.8%) patients with ICI-induced colitis and 3/157 (1.9%) controls (*P* < 0.001). Patients with anti-integrin αvβ6 autoantibodies had significantly more typical UC endoscopic features than those without the autoantibodies (*P* < 0.001). Anti-integrin αvβ6 autoantibodies in ICI-induced colitis patients were associated with grade ≥3 colitis (*P* = 0.001) and steroid resistance (*P* = 0.005). Anti-integrin αvβ6 autoantibody titers correlated with ICI-induced colitis disease activity. Anti-integrin αvβ6 autoantibodies of ICI-induced colitis exhibited similar characteristics to those of UC.

**Conclusions:**

Anti-integrin αvβ6 autoantibodies may serve as potential biomarkers for the diagnosis, classification, risk management, and monitoring the disease activity, of ICI-induced colitis.

## Introduction

Immune checkpoint inhibitors (ICIs) have revolutionized tumor treatment strategies, showing significant efficacy against diverse malignancies [1]. However, inhibiting immune checkpoints can cause inflammation in various organs, leading to immune-related adverse events (irAEs) that resemble autoimmune diseases [2, 3]. With the increasing use of ICIs in cancer treatment regimens, the incidence of newly diagnosed irAEs continues to rise. ICI-induced colitis, the most common form of gastrointestinal (GI) irAEs, shows a broad spectrum of disease severity, endoscopic findings, and therapeutic responses [4, 5]. Rapid diagnosis followed by adequate immunosuppression with corticosteroids is essential for the treatment of moderate to severe ICI-induced colitis [4, 5]. However, an early switch to stronger immunosuppressive therapies, such as infliximab and vedolizumab, is required in cases with steroid-refractory ICI-induced colitis, which can cause life-threatening complications including colorectal perforation and subsequently, unfavorable patient outcomes [4, 5]. Therefore, reliable and objective biomarkers for the diagnosis, monitoring, and risk management of ICI-induced colitis are crucial for achieving favorable patient outcomes; however, thus far, no specific biomarkers for this condition have been identified.

Some studies have demonstrated the endoscopic and histopathological similarities between ICI-induced colitis and inflammatory bowel disease (IBD) [6–11]. However, whether IBD-like ICI-induced colitis has a similar pathophysiology to IBD remains to be elucidated. We recently identified anti-integrin αvβ6 autoantibodies in Japanese patients with ulcerative colitis (UC), the most common type of IBD [12]. Remarkably, the sensitivity and specificity of these autoantibodies for UC are both >90% and those titers are correlated with the disease activity of UC [12]. These results are replicated in studies in United States and Sweden by other groups [13, 14], further supporting the reliability of anti-integrin αvβ6 autoantibodies as a diagnostic and disease activity marker for UC.

Integrins are a major family of heterodimeric cell adhesion receptors comprising 18 α- and 8 β- subunits that form 24 distinct integrin heterodimers [15]. Among them, integrin αvβ6 has been reported to be present in the intestine, bind to extracellular matrix proteins such as fibronectin [16], and play an important role in maintaining epithelial barrier function [17]. Furthermore, integrin αvβ6 is also widely expressed in various types of cancer [18].

In this study, given the similarities between ICI-induced colitis and IBD, we examined whether patients with ICI-induced colitis also possess anti-integrin αvβ6 autoantibodies, and found that certain patients with ICI-induced colitis have these autoantibodies. We also investigated the clinical features of such patients. This study will provide insight into the pathogenesis and heterogeneity of ICI-induced colitis.

## Materials and methods

### Patients

This was a retrospective study targeting patients with ICI-induced colitis who had undergone colonoscopy at the onset of the disease at Kyoto University Hospital, affiliated hospitals, and Kindai University Faculty of Medicine between April 2018 and April 2023. All but one patient (Case 1) was treated with only ICI treatment. ICI-induced colitis was defined as diarrhea or bloody stools following ICI administration and/or with histological evaluation [5]. Patients with preexisting IBD and infectious enteritis caused by pathogenic microorganisms, such as *Clostridioides difficile*, *Campylobacter jejuni*, and *Cytomegalovirus*, were excluded. The severity of ICI-induced colitis was evaluated using the Common Terminology Criteria for Adverse Events (CTCAE) version 5.0 [19]. The clinical characteristics of each patient with ICI-induced colitis such as age, sex, ICI medication, cancer type, time from ICI initiation to onset, CTCAE grade of diarrhea and colitis, treatment for irAE, comorbidities, other irAEs, and prognosis are shown in Supplementary Table S1 and summarized in Supplementary Table S2. Serum samples were collected from each patient at the time of diagnosis. Among them, serial blood samples were available in four patients with ICI-induced colitis (Case 1, 10, 14, and 20), and the disease activity of the four patients was evaluated using the full or partial Mayo score [20]. Serum samples were also collected from 39 patients with irAEs in other organs, 77 patients with cancer but without irAEs, and 41 healthy volunteers as controls (Supplementary Table S3). Sera from 12 patients with UC were used to compare autoantibody characteristics between ICI-induced colitis and UC (Supplementary Table S4). All serum samples were stored at −80 °C until assayed.

The study was performed in accordance with the Declaration of Helsinki and was approved by the Ethics Committee of Kyoto University Graduate School and Faculty of Medicine (protocol number: R1004). All participants provided written informed consent.

### Analysis of endoscopic findings

Two experienced endoscopists (M.O. and H.K.) who were blinded to the anti-integrin αvβ6 antibody titer evaluated endoscopic findings. Both endoscopists majored in IBD and had over 10 years of work experience. The endoscopic findings of the patients were classified into typical UC findings as described in Supplementary Table S5 [21]. The presence or absence of the endoscopic findings in each patient was scored as 1 or 0, respectively, and the total score was calculated (Supplementary Table S6). The average of the scores assigned by the two endoscopists was used for further analysis.

### Enzyme-linked immunosorbent assay

Recombinant human integrin heterodimers were purchased from ACROBiosystems (Newark, DE, USA), and recombinant human integrin β6 monomer was kindly provided by Medical and Biological Laboratories (Tokyo, Japan) (Supplementary Table S7). To detect serum immunoglobulin G (IgG) antibodies against integrins, we used an enzyme-linked immunosorbent assay (ELISA) Starter Accessory kit (E101, Bethyl Laboratories, Montgomery, TX, USA) according to the manufacturer’s instructions. Briefly, microtiter plates were coated with 100 μL of the recombinant proteins (2 μg/mL) overnight at 4 °C, blocked, and incubated for 60 min with 100 µL of diluted serum (1:100) at room temperature. After five washes with wash solution, the plates were incubated for 60 min with 100 µL rabbit anti-human IgG antibody conjugated with horseradish peroxidase (HRP) (1:50,000; ab6759, Abcam, Cambridge, UK) at room temperature. After additional five washes with wash solution, the bound reactants were identified by incubating with 3,3′,5,5′-tetramethylbenzidine (TMB) for 7 min at room temperature. The absorbance was measured at 450 nm. ELISA was performed with MgCl_2_ and CaCl_2_ (1 mM each) [12].

The subclasses of the autoantibodies were identified using anti-human IgG1, IgG2, IgG3, and IgG4 secondary antibodies conjugated with HRP (1:2,000; BS-AP006, BS-AP007, BS-AP008, and BS-AP009, respectively; The Binding Site, Birmingham, UK). In addition, the autoantibody isotypes were evaluated using anti-human IgA, IgM, and IgE secondary antibodies conjugated with HRP (1:50,000 A80-102P, 1:100,000 A80-100P, and 1:1,000 A80-108P, respectively; Bethyl Laboratories).

To investigate whether the Arg-Gly-Asp (RGD) peptide inhibited the binding of IgG obtained from patients with ICI-induced colitis with anti-integrin αvβ6 autoantibodies against integrin αvβ6, we added the Arg-Gly-Asp-Ser (RGDS) peptide (A9041, Sigma-Aldrich, St. Louis, MO, USA) or the control peptide Arg-Gly-Glu-Ser (RGES) (A5686, Sigma-Aldrich) to the serum at concentrations of 25 and 100 μg/mL before incubation.

### Isolation of human immunoglobulin G

IgG was isolated from the sera of patients and healthy volunteers using Ab-Rapid SPiN EX (P-014; ProteNova, Higashikagawa, Japan) and stored at −30 °C. In our previous study, the rate of IgG recovery from the sera was verified to be >90% [12, 22].

### Solid-phase integrin αvβ6 binding assay

A solid-phase integrin αvβ6 binding assay was performed following a previously described method with minor modifications [12, 23]. Briefly, a 96-well microtiter plate was coated with 100 µL/well integrin αvβ6 (2 µg/mL) overnight at 4 °C, blocked, and incubated with 100 µL of diluted patient or control IgG (1:10) for 60 min at room temperature. After five washes with wash solution, the plates were incubated with 100 µL fibronectin (2 µg/mL; FC010, Millipore Sigma, Burlington, MA, USA) for 60 min at room temperature. After five washes with wash solution, an anti-fibronectin antibody (1:5,000; ab2413, Abcam) was added, followed by incubation for 60 min at room temperature. Afterward, the plates were washed with wash solution five times, and an anti-rabbit IgG HRP-conjugated secondary antibody (1:10,000; A27036, Thermo Fisher Scientific, MA, USA) was added, followed by incubation for 60 min at room temperature. The plates were washed (five times with wash solution), incubated with TMB for 10 min at room temperature, and bound reactants were identified. The absorbance was measured at 450 nm. A solid-phase integrin αvβ6 binding assay was performed in the presence of MgCl_2_ and CaCl_2_ (1 mM each).

The inhibition rate was calculated as follows: [(control optical density (OD) – sample OD)/control OD]. The control OD was measured by coating the control wells with integrin αvβ6 and incubating with fibronectin in the absence of patient or control IgG.

### Immunohistochemical analysis

The immunohistochemical analysis was performed according to standard procedures for human tissue sections. Because integrin β6 forms a dimer only with integrin αv, whereas, αv can dimerize with other β subunits, including β1, β3, β5, and β8 [24], antibodies against integrin β6 were used to detect integrin αvβ6 expression. Antigen retrieval was performed on sections by incubating them in citrate buffer (pH 6.0) for 20 min at 121 °C in an autoclave before incubating overnight at 4 °C with the antibodies against integrin β6 (1:500; HPA023626, Sigma-Aldrich). Liquid DAB+ Substrate Chromogen System (K3468, Dako, Santa Clara, CA, USA) was used for staining. Detection times were equally standardized for all sections. Staining intensity of integrin β6 was graded as either 0, 1+, 2+, or 3+. The H-score was calculated using the following formula: [1 × (% cells 1+) + 2 × (% cells 2+) + 3 × (% cells 3+)].

### Statistical analysis

Fisher’s exact test was performed to evaluate categorical variables. Continuous variables were compared using Mann–Whitney U tests. Intraclass correlation coefficient (ICC) was used to determine the reliability of the endoscopic scores determined by the two endoscopists. GraphPad Prism Version 9 (GraphPad Software, San Diego, CA, USA) and Stata 18 (StataCorp, College Station, TX, USA) were used for statistical analysis. Two-tailed *P* < 0.05 was considered statistically significant.

## Results

### Clinical characteristics of patients with ICI-induced colitis

Twenty-six patients clinically diagnosed with ICI-induced colitis and undergoing colonoscopy at the onset of the disease were enrolled in the study. The baseline clinical characteristics of the patients with ICI-induced colitis are summarized in Supplementary Table S2. The median age of patients at the onset of ICI-induced colitis was 69.5 years (range: 47–82 years). There were 18 men and eight women. Eighteen (69.2%) patients were treated with PD-1 inhibitors, five (19.2%) with CTLA-4 and PD-1 inhibitors, and three (11.5%) with PD-L1 inhibitors. The median time from treatment initiation to ICI-induced colitis onset was 108 days (range: 14–837 days). Furthermore, 12 (46.2%) patients had grade ≥3 diarrhea, while seven (26.9%) developed grade ≥3 colitis based on symptoms. Fifteen (57.7%) patients were treated with steroids alone, whereas four (15.4%) were treated with steroids plus infliximab. Seven (26.9%) patients improved with conservative treatment alone. With regard to prognosis, 10 (38.5%) patients had died because of the progression of primary malignancies at the time of analysis (median follow-up time of 25 months [range: 4–61 months]).

### Detection of anti‐integrin αvβ6 autoantibodies in patients with ICI-induced colitis

**Fig. 1:**
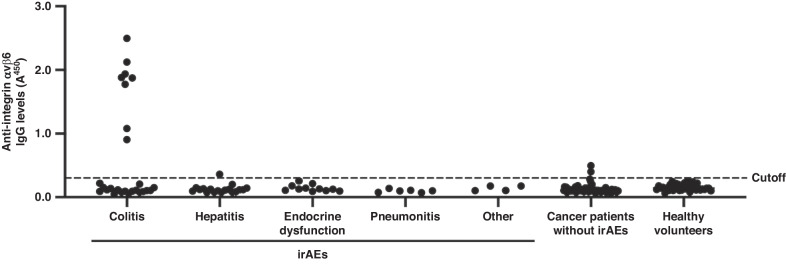
Detection of anti-integrin αvβ6 autoantibodies in serum samples of patients with ICI-induced colitis. Serum IgG antibodies against integrin αvβ6 were quantified using ELISA. The sera of 26 patients with ICI-induced colitis, 39 ICI-treated patients with other types of irAEs (18 with hepatitis, 11 with endocrine dysfunction, 6 with pneumonitis, 4 with other irAEs), 77 patients with cancer without irAEs (16 with colon cancer, 15 with non-small cell lung cancer, 15 with gastric cancer, 15 with bile duct cancer, 12 with pancreatic cancer, 4 with melanoma), and 41 healthy volunteers were examined (Supplementary Table S1, Supplementary Table S3). IgG antibodies against integrin αvβ6 were identified in 8/26 (30.8%) patients with ICI-induced colitis and 3/157 (1.9%) controls. The cutoff OD value, defined as the mean plus three SDs of sera from the healthy volunteers, is indicated using a dashed line. The experiment was repeated independently twice with similar results. ELISA enzyme-linked immunosorbent assay, ICI immune checkpoint inhibitor, IgG immunoglobulin G, irAEs immune-related adverse events, OD optical density, SD standard deviation.

We performed ELISA to identify anti-integrin αvβ6 autoantibodies in patients with ICI-induced colitis. Based on a cutoff OD of the mean plus three standard deviations of healthy volunteer sera, eight (30.8%; 8/26) patients with ICI-induced colitis were identified positive for IgG autoantibodies against integrin αvβ6. In contrast, similar IgG autoantibodies were identified in 3 controls (1.9%; 3/157) (Fig. 1). Anti-integrin αvβ6 autoantibodies had a sensitivity and specificity of 30.8% and 98.1%, respectively, in patients with ICI-induced colitis (*P* < 0.001). The specificity of anti-integrin αvβ6 autoantibodies for patients with ICI-induced colitis compared with that for patients with other irAEs or those with cancer without irAEs was 97.4% (*P* = 0.002) and 97.4% (*P* < 0.001), respectively. Eight out of 21 (38.1%) ICI-induced colitis patients treated with PD-1/PD-L1 inhibitor monotherapy were positive for the autoantibodies, while all five ICI-induced colitis patients treated with combination of CTLA-4 and PD-1 inhibitors were negative for the autoantibodies (Table 1).

**Table 1:** Comparison between anti-integrin αvβ6 antibody-positive and anti-integrin αvβ6 antibody-negative patients with ICI-induced colitis.

	Anti-integrin αvβ6 antibodies		
	Positive	Negative	*P* value
**Number of patients,** ***n***	8	18	
Median age, years (range)	69 (57–82)	70.5 (47–80)	0.404
Sex			
Male, *n* (%)	5 (62.5)	13 (72.2)	0.667
Female, *n* (%)	3 (37.5)	5 (27.8)	
**Endoscopic findings**			
Number of typical UC findings (median)	4.5	1.5	<0.001
**Cancer type,** ***n*** **(%)**			
Non-small cell lung cancer	2 (25)	6 (33.3)	0.815
Melanoma	3 (37.5)	4 (22.2)	
Kidney cancer	1 (12.5)	4 (22.2)	
Esophageal cancer	1 (12.5)	1 (5.6)	
Peritoneal cancer	1 (12.5)	0 (0)	
Cancer of unknown primary	0 (0)	1 (5.6)	
Malignant pleural mesothelioma	0 (0)	1 (5.6)	
Bladder cancer	0 (0)	1 (5.6)	
**ICI medication,** ***n*** **(%)**			
Pembrolizumab	4 (50)	6 (33.3)	0.429
Nivolumab	3 (37.5)	5 (27.8)	
Nivolumab and ipilimumab	0 (0)	5 (27.8)	
Atezolizumab	1 (12.5)	1 (5.6)	
Durvalumab	0 (0)	1 (5.6)	
**Grade** ≥ **3 adverse events,** ***n*** **(%)**			
Diarrhea	5 (62.5)	7 (38.9)	0.401
Colitis	6 (75)	1 (5.6)	0.001
**Treatment,** ***n*** **(%)**			
Steroid alone	3 (37.5)	12 (66.7)	0.218
Conservative treatment	1 (12.5)	6 (33.3)	0.375
Steroid/infliximab	4 (50)	0 (0)	0.005
**Prognosis,** ***n*** **(%)**			
Alive	4 (50)	12 (66.7)	0.664
Dead	4 (50)	6 (33.3)	

Severity of diarrhea and colitis was assessed according to CTCAE version 5.0 [19].

### UC-like endoscopic findings and severe symptoms for patients with ICI-induced colitis possessing anti-integrin αvβ6 autoantibodies

**Fig. 2:**
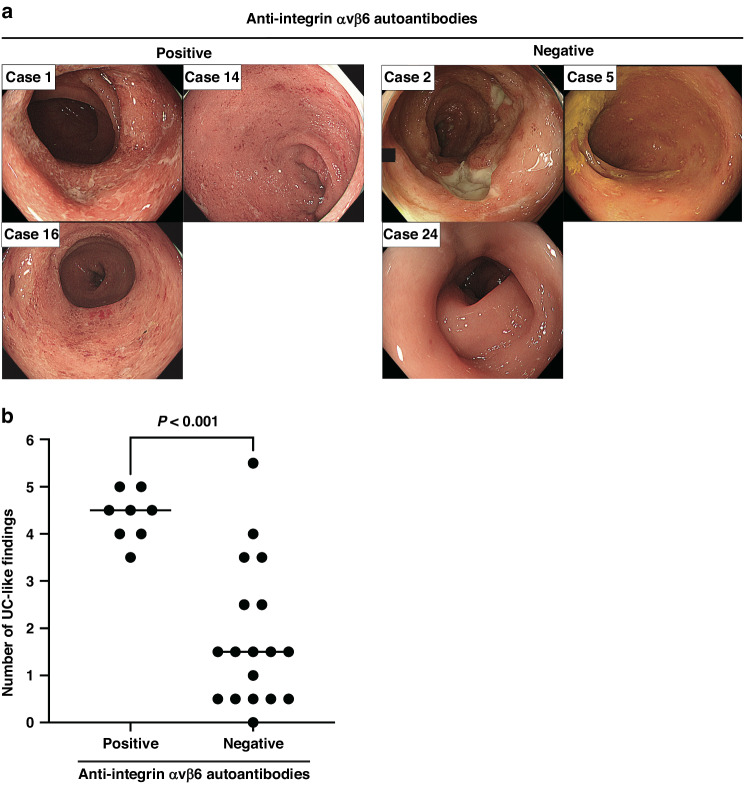
Differences in endoscopic findings between patients with ICI-induced colitis with and without anti-integrin αvβ6 autoantibodies. **a** Endoscopic images of patients with ICI-induced colitis with anti-integrin αvβ6 autoantibodies revealed findings similar to those of UC, including erythema, granularity, decreased vascular pattern, bleeding, and ulcer formation (Supplementary Table S5). In contrast, negative cases showed findings atypical of UC, such as punched ulcers. All patients underwent colonoscopy at the onset of the disease. **b** The average total number of typical UC findings assessed by two endoscopists was significantly higher in patients with ICI-induced colitis with anti-integrin αvβ6 autoantibodies compared to that in those without autoantibodies (median scores, 4.5 vs. 1.5; *P* < 0.001). The horizontal lines represent the median. ICI immune checkpoint inhibitor, UC ulcerative colitis.

Next, we compared endoscopic findings and clinical characteristics between patients with ICI-induced colitis with or without anti-integrin αvβ6 autoantibodies. Independent blinded assessment revealed that the endoscopic findings were more similar between patients with autoantibodies and those with UC than those in patients without autoantibodies (Fig. 2a). Furthermore, the average score of typical UC findings was significantly higher in patients with ICI-induced colitis with anti-integrin αvβ6 autoantibodies than that in those without the autoantibodies (median scores, 4.5 vs. 1.5; *P* < 0.001) (Fig. 2b, Table 1, Supplementary Table S5, Supplementary Table S6). The ICC of endoscopic scores estimated by the two endoscopists was 0.85 (95% confidence interval, 0.56–0.94). Anti-integrin αvβ6 autoantibodies in patients with ICI-induced colitis were significantly associated with CTCAE grade ≥3 colitis (6/8, 75% vs. 1/18, 5.6%; *P* = 0.001; Table 1). In addition to steroids, infliximab treatment was required only for the patients with anti-integrin αvβ6 autoantibodies (4/8, 50% vs. 0/18, 0%; *P* = 0.005; Table 1). These findings suggest that anti-integrin αvβ6 autoantibodies are potential markers to identify ICI-induced colitis harboring UC-like endoscopic findings and severe symptoms that require stronger immunosuppressive therapy.

### Correlation between anti-integrin αvβ6 autoantibody titers and disease activity

**Fig. 3:**
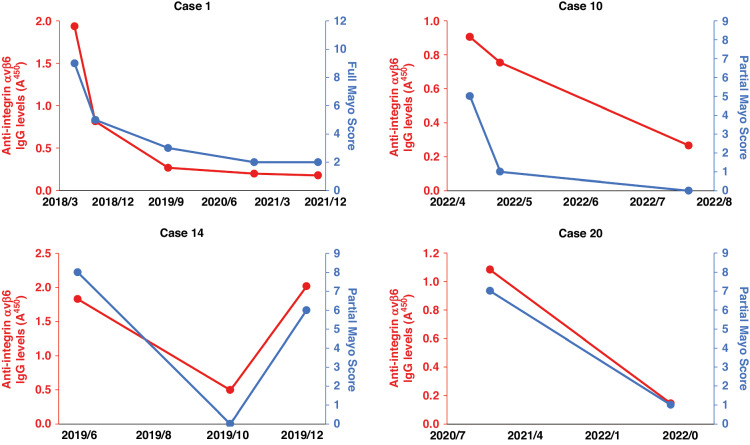
Correlation between anti-Integrin αvβ6 autoantibody titers and disease activity in patients with ICI-induced colitis with anti-integrin αvβ6 autoantibodies. Serial blood samples from four patients with ICI-induced colitis with anti-integrin αvβ6 autoantibodies (Case 1, 10, 14, and 20) were used. Changes in autoantibody titers against integrin αvβ6 in ICI-induced colitis patients with anti-integrin αvβ6 autoantibodies correlated with the changes in the full or partial Mayo score. The left y-axis and the red points represent the OD values of anti-integrin αvβ6 serum IgG levels, while the right y-axis and the blue points represent the full or partial Mayo score. ICI immune checkpoint inhibitor, IgG immunoglobulin G, OD optical density.

Changes in anti-integrin αvβ6 antibody titers were investigated using serially collected serum samples from four patients with ICI-induced colitis who were positive for the autoantibodies. The full or partial Mayo score corresponded with changes in antibody titers against integrin αvβ6 in patients with ICI-induced colitis (Fig. 3). These findings suggest that anti-integrin αvβ6 autoantibody titers reflect disease activity in patients with ICI-induced colitis resembling UC.

### Similar antibody characteristics between patients with ICI-induced colitis and those with UC in subclass, isotype, epitope, and functions

We investigated the characteristics of anti-integrin αvβ6 autoantibodies in the patients with ICI-induced colitis and compared them to those in UC. We previously found that anti-integrin αvβ6 autoantibodies in UC patients show a predominance of the IgG1 subclass and IgA isotype [12]. In this study, ELISA results showed that seven and eight of the eight patients with ICI-induced colitis positive for the antibody had IgG1 and IgA autoantibodies, respectively (Supplementary Fig. S1). These data in patients with ICI-induced colitis were similar to those in patients with UC.

We also checked the similarity of epitope of anti-integrin αvβ6 autoantibodies between ICI-induced colitis and UC patients. We examined whether the integrin αvβ6 autoantibodies might recognize integrin αvβ6 dimer, or monomers of integrin αv or integrin β6. We were able to obtain a monomer of integrin β6 that was verified by ELISA (Supplementary Fig. S2). However, we could not obtain a monomer of integrin αv. Therefore, we assayed the serum samples for autoantibodies against integrin αvβ1, αvβ3, αvβ5, or αvβ8 to assess the reactivity with integrin αv. Six (75%) patients with anti-integrin αvβ6 autoantibodies had anti-integrin αvβ3 autoantibodies similar to patients with UC [12], but none had autoantibodies against other αv-containing integrins and monomer of integrin β6 (Supplementary Fig. S3). These results suggest that the anti-integrin αvβ6 autoantibodies of both patients with ICI-induced colitis and UC bind to dimeric conformation of integrin αvβ6.

**Fig. 4:**
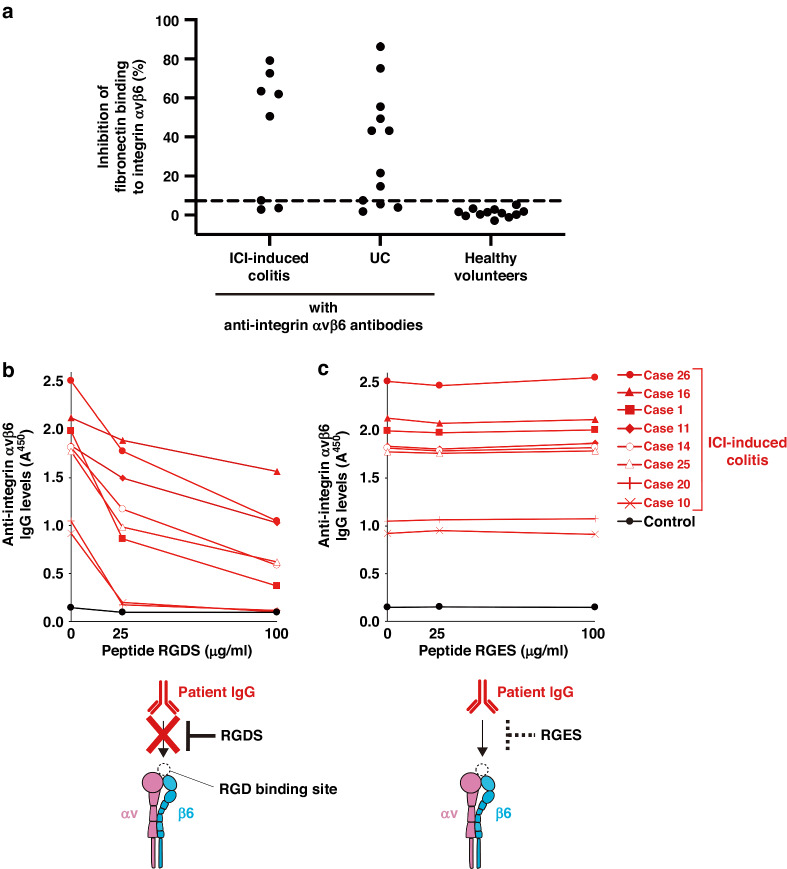
Blocking of integrin αvβ6-fibronectin binding by IgG from patients with ICI-induced colitis with anti-integrin αvβ6 autoantibodies. **a** Inhibition of integrin αvβ6-fibronectin binding by IgG of patients with ICI-induced colitis with anti-integrin αvβ6 autoantibodies in the solid-phase binding assay. The cutoff OD value, defined as the mean plus three SDs of IgG from healthy volunteers, is indicated using a dashed line. IgG from six of eight (75%) patients with ICI-induced colitis with anti-integrin αvβ6 autoantibodies blocked integrin αvβ6-fibronectin binding. However, no control IgG showed blocking activity. **b**, **c** Peptide RGDS, but not RGES, dose-dependently impaired the binding of IgG of patients with ICI-induced colitis with anti-integrin αvβ6 autoantibodies against integrin αvβ6. The RGDS and RGES peptides represented the RGD and RGE motifs, respectively. The experiments were repeated independently twice with similar results. ICI immune checkpoint inhibitor, IgG immunoglobulin G, OD optical density, RGD Arg-Gly-Asp, RGDS Arg-Gly-Asp-Ser, RGE Arg-Gly-Glu, RGES Arg-Gly-Glu-Ser, SD standard deviation.

As shown in Supplementary Fig. S4a, integrin αvβ6 binds to its ligands, such as fibronectin, through the RGD tripeptide motif [25]. In this study, solid-phase binding assay revealed that IgG from six of eight (75%) patients with ICI-induced colitis with anti-integrin αvβ6 autoantibodies blocked integrin αvβ6-fibronectin binding (Fig. 4a, Supplementary Fig. S4b). These findings are consistent with those of our previous study, which showed that IgG purified from patients with UC blocked integrin αvβ6-fibronectin binding [12].

Furthermore, similar to our previous study, we showed that RGDS peptides inhibited the binding of IgG of patients with ICI-induced colitis with anti-integrin αvβ6 autoantibodies to integrin αvβ6 in a dose-dependent manner (Fig. 4b, Supplementary Fig. S4c). However, RGES peptides (control) did not inhibit this binding (Fig. 4c). These results suggest that like that in patients with UC, anti-integrin αvβ6 autoantibodies in patients with ICI-induced colitis bind to the RGD binding site of integrin αvβ6.

Altogether, these findings suggest that anti-integrin αvβ6 autoantibodies in patients with ICI-induced colitis show similar characteristics to those in patients with UC [12].

### Expression of integrin αvβ6 in tumor tissues of the patients with ICI-induced colitis with anti‐integrin αvβ6 autoantibodies

**Fig. 5:**
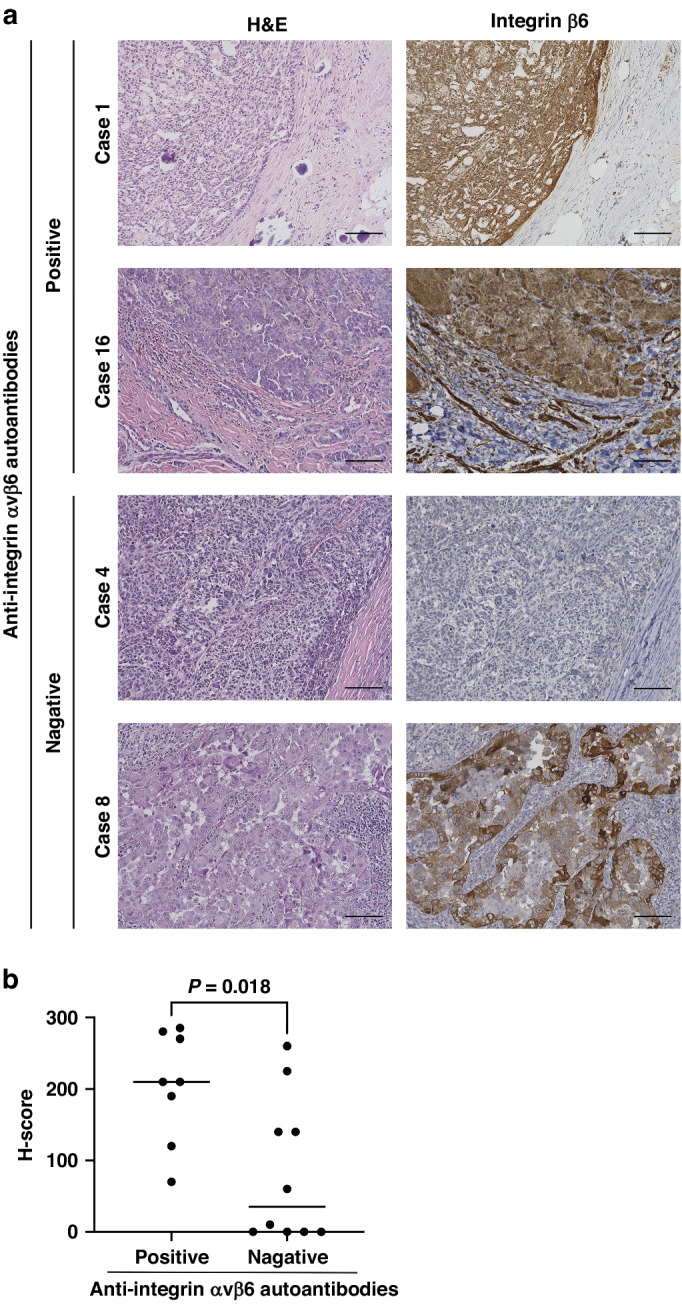
Expression of integrin αvβ6 in tumor tissues of the patients with ICI-induced colitis with and without anti‐integrin αvβ6 autoantibodies. **a** Representative H&E staining and immunohistochemical staining of integrin β6 in tumors of ICI-induced colitis patients with or without integrin αvβ6 autoantibodies. Scale bars, 100 µm. **b** H-score for integrin β6 immunostaining in tumors of patients with ICI-induced colitis with or without integrin αvβ6 autoantibodies. Horizontal lines indicate median values. The experiments were repeated independently twice with similar results. H&E hematoxylin-eosin, ICI immune checkpoint inhibitor.

Next, we investigated whether the primary tumors of patients with ICI-induced colitis with anti-integrin αvβ6 autoantibodies express integrin αvβ6, given that production of autoantibodies may be induced by the immune reaction to primary tumors. Immunohistochemical analysis revealed that all eight patients with ICI-induced colitis with anti-integrin αvβ6 autoantibodies had tumors that expressed integrin αvβ6. By contrast, integrin αvβ6 expression was less frequent in patients without anti-integrin αvβ6 autoantibodies (8/8, 100% vs. 5/10, 50%; *P* = 0.036; Fig. 5a). Moreover, the H-score for integrin β6 immunostaining was significantly higher in the tumors of patients with ICI-induced colitis with anti-integrin αvβ6 autoantibodies than that in those without the autoantibodies (median scores, 210 vs. 35; *P* = 0.018; Fig. 5b). These findings may indicate a causal association between integrin αvβ6 expression in the primary tumor and anti-integrin αvβ6 autoantibodies in the serum.

## Discussion

In this study, we found that a part of ICI-induced colitis patients possessed anti-integrin αvβ6 autoantibodies and such patients exhibited endoscopically UC-like colitis with severe symptoms that required intensive therapy. The titers of anti-integrin αvβ6 autoantibodies correlated with disease activity of ICI-induced colitis. Anti-integrin αvβ6 autoantibodies in patients with ICI-induced colitis had similar characteristics to those in patients with UC. Moreover, immunohistochemistry analysis revealed integrin αvβ6 expression in tumor tissues of all patients with anti-integrin αvβ6 autoantibodies.

Considering that the current diagnosis of ICI-induced colitis is primarily based on non-specific observations rather than on specific diagnostic markers [4, 5], it is sometimes difficult to differentiate ICI-induced colitis from other types of colitis. At present, the gold standard for diagnosing and monitoring ICI-induced colitis is colonoscopy [4, 5]. Several studies have revealed that endoscopically confirmed ulceration or pancolitis indicates steroid-refractory ICI-induced colitis that requires early intensive immunosuppression [9, 26–28]. However, although monitoring by repeat colonoscopy is recommended for individuals who are refractory to immunosuppressive agents [4], frequent colonoscopy can be a physical burden on cancer patients. Blood tests, including complete blood count, comprehensive metabolic panel, C-reactive protein (CRP), and thyroid-stimulating hormone, are also recommended for noninvasive assessment of ICI-induced colitis [4, 5]. However, these factors are not specific to ICI-induced colitis, and moreover, it has been reported that the biochemical parameters, including CRP, albumin, and hemoglobin, did not correlate with the severity of ICI-induced colitis [28]. Several guidelines have suggested fecal calprotectin and lactoferrin as alternatives or adjuncts to endoscopic evaluation for monitoring ICI-induced colitis [4, 5]. However, fecal calprotectin levels could also be elevated in patients with malignant tumors in the GI tract [29, 30]. These studies highlight the challenges associated with the diagnosis and monitoring of ICI-induced colitis.

In this study, we showed that a part of the patients with ICI-induced colitis had anti-integrin αvβ6 autoantibodies. Based on these autoantibodies, an accurate diagnosis of ICI-induced colitis may help facilitate the early implementation of the appropriate treatment for ICI-induced colitis. In addition, patients with ICI-induced colitis with anti-integrin αvβ6 autoantibodies exhibited more severe symptoms than those without the autoantibodies. These findings suggest that anti-integrin αvβ6 autoantibodies may serve as a potential biomarker to identify life-threatening ICI-induced colitis, which requires more intensive immunosuppressive therapy. Together, this noninvasive marker may be useful in clinical decisions and monitoring the disease activity during therapy.

As for characteristics of anti-integrin αvβ6 autoantibodies, we found that both IgG subclass and immunoglobulin isotype of anti-integrin αvβ6 autoantibodies in ICI-induced colitis patients were similar to those identified in UC patients. Moreover, anti-integrin αvβ6 autoantibodies bound to the dimeric conformation of integrin αvβ6 and blocked integrin αvβ6-fibronectin binding via the RGD motif. These characteristics are also similar to those of anti-integrin αvβ6 autoantibodies in patients with UC [12]. These data suggest that the UC and ICI-induced colitis with anti-integrin αvβ6 autoantibodies may share a common pathophysiology, although it remains unclear whether anti-integrin αvβ6 autoantibodies have a pathological role. Nonetheless, using the same treatment strategy as UC, such as infliximab and vedolizumab, to treat patients with ICI-induced colitis with autoantibody appears reasonable. In addition, given that the pathogenesis and clinical manifestations of ICI-induced colitis are considered to be heterogeneous, autoantigens in ICI-induced colitis patients without anti-integrin αvβ6 autoantibodies, whose clinical manifestations are similar to Crohn’s disease, or microscopic colitis, may be different from integrin αvβ6. Therefore, the measurement of anti-integrin αvβ6 autoantibodies might contribute to the classification of the ICI-induced colitis.

Interestingly, we found that integrin αvβ6 expression was observed in all tumors obtained from patients positive for anti‐integrin αvβ6 autoantibodies. In contrast, only half of the autoantibody-negative cases exhibited such expression. These findings indicate that integrin αvβ6 in the primary tumor may be related to anti‐integrin αvβ6 autoantibody production. However, further investigations are required in this regard, given the relatively frequent (50%) expression of integrin αvβ6 in the tumors of patients without anti-integrin αvβ6 autoantibodies.

There are some limitations of this study. The sample size used in this research was relatively small. Furthermore, this study did not include patients with mild ICI-induced colitis who had not undergone colonoscopy. Given the nature of the retrospective study, it should also be recognized that there is substantial heterogeneity in the clinical evaluation and management of ICI-induced colitis among institutions. A prospective study with a large number of patients is therefore needed to confirm our present findings. It may also be possible that patients with ICI-induced colitis with anti-integrin αvβ6 autoantibodies had predisposing factors for UC before ICI treatment. Therefore, a future study that measures antibody titers at baseline before ICI administration is necessary.

In conclusion, this study revealed that anti-integrin αvβ6 autoantibodies could be a potential biomarker for diagnosis and assessment of disease activity of ICI-induced colitis. Our findings suggest the importance of these autoantibodies for the classification and risk management of ICI-induced colitis. Further large-scale studies are warranted to confirm that anti-integrin αvβ6 autoantibodies are a useful biomarker for high-risk ICI-induced colitis.

### Supplementary information

Revised Supplementary Materials

## Data Availability

The data supporting the findings of this study are available from the corresponding author upon reasonable request.
