# Supplementary material for: Anti-integrin αvβ6 autoantibodies are a potential biomarker for ulcerative colitis-like immune checkpoint inhibitor-induced colitis
Source: Br J Cancer. 2024 Mar 9;130(9):1552–60. doi: 10.1038/s41416-024-02647-1 (PMC11058246; doi:10.1038/s41416-024-02647-1)
Supplement: Supplementary file 1 — Revised Supplementary Materials [file 41416_2024_2647_MOESM1_ESM.docx]

**Supplementary Materials**

**Supplementary Figures**

**Supplementary Figure S1.** **Anti-integrin αvβ6** **autoantibody IgG subclasses and isotypes in patients with ICI-induced colitis.**

(A) The serum samples were incubated with integrin αvβ6, followed by incubation with HRP-conjugated antibodies specific for each human IgG subclass. Seven (87.5%) seven (87.5%), three (37.5%), and two (25%) ICI-induced colitis patients with anti-integrin αvβ6 autoantibodies had antibodies against IgG1, IgG2, IgG3, and IgG4 antibodies, respectively. (B) Serum samples were incubated with integrin αvβ6, followed by incubation with HRP-conjugated antibodies specific for human IgA, IgM, or IgE. Eight (100%), zero (0%), and zero (0%) ICI-induced colitis patients with anti-integrin αvβ6 autoantibodies had IgA, IgM, and IgE antibodies, respectively. The dashed line indicates the cutoff OD value, defined as the mean plus three SDs of the sera of six healthy volunteers. The experiments were repeated independently twice with similar results.

Abbreviations: HRP, horseradish peroxidase; ICI, immune checkpoint inhibitors; IgG, immunoglobulin G; OD, optical density; SD, standard deviation.

**Supplementary Figure S2. Verification of Integrin β6 as a monomer.**

We checked the quality of integrin β6 using ELISA. Briefly, microtiter plates were coated with 100 μL of 2 μg/mL of integrin αvβ6, β6, and α8β1, overnight at 4 °C and blocked at room temperature. After five washes with wash solution, the plates were incubated with 100 µL of rabbit anti-integrin αv antibody (left) or rabbit anti-integrin β6 antibody (right) (1:5,000; ab179475 or ab197672, respectively, Abcam) for 60 min at room temperature. After five washes with wash solution, the plates were incubated with 100 µL of anti-rabbit IgG HRP-conjugated secondary antibody (1:5,000; 31458, Thermo Fisher Scientific) for 60 min at room temperature. After another five washes with wash solution, the bound reactants were detected by incubating with TMB for 10 min at room temperature. The absorbance of the samples was measured at 450 nm. Both antibody preparations were detected by integrin αvβ6; however, only the antibodies against integrin β6 detected integrin β6. Integrin α8β1, examined as a negative control, was not detected by either antibody preparation.

Abbreviations: ELISA, enzyme-linked immunosorbent assay; HRP, horseradish peroxidase; IgG, immunoglobulin G; TMB, 3,3′,5,5′-tetramethylbenzidine.

**Supplementary Figure S3.** **Non-binding of anti-integrin αvβ6 autoantibodies to integrin αv and β6 in patients with ICI-induced colitis.**

Serum IgG antibodies against integrin αvβ1, integrin αvβ3, integrin αvβ5, and integrin αvβ8 (assessed for recognition of integrin αv), and integrin β6 were quantified using ELISA. Six of the eight ICI-induced colitis patients with anti-integrin αvβ6 antibodies exhibited anti-integrin αvβ3 autoantibodies, which was comparable to the case in UC patients [1], but none had autoantibodies against other integrins, including integrin αvβ1, αvβ5, αvβ8, and β6. The dashed line indicates the cutoff OD value, defined as the mean plus three SDs of the sera of six healthy volunteers. The experiments were repeated independently twice with similar results.

Abbreviations: ELISA, enzyme-linked immunosorbent assay; ICI, immune checkpoint inhibitor; OD, optical density; SD, standard deviation; UC, ulcerative colitis.

**Supplementary Figure S4.** **Binding of integrin αvβ6 to fibronectin through RGD sequence motif recognition and the inhibition of its activity by autoantibodies against integrin αvβ6.**

(A) Schematic illustration of the binding of integrin αvβ6 to fibronectin by recognizing the RGD sequence motif. (B) Schematic representation of the solid-phase binding assay. (C) The binding assay for investigating whether the RGD peptide inhibits the binding of IgG with anti-integrin αvβ6 autoantibodies against integrin αvβ6 in ICI-induced colitis patients.

Abbreviations: HRP, horseradish peroxidase; IgG, immunoglobulin G; RGD, Arg-Gly-Asp; RGDS, Arg-Gly-Asp-Ser; RGES, Arg-Gly-Glu-Ser; TMB, 3,3′,5,5′-tetramethylbenzidine.

**Supplementary Tables**

**Supplementary Table S1. Clinical information of patients with ICI-induced colitis**

| **Case** | **Age**  **(years)** | **Sex** | **ICI medication** | **Cancer type** | **Time from ICI initiation to onset (days)** | **CTCAE**  **grade (diarrhea)** | **CTCAE**  **grade (colitis)** | **Treatment** | **Comorbidities** | **Other irAEs** | **Prognosis**  **(months)** |
| --- | --- | --- | --- | --- | --- | --- | --- | --- | --- | --- | --- |
| 1 | 57 | F | Atezolizumab*^a^* | Peritoneal cancer | 138 | 2 | 3 | Steroid | - | - | Alive  (61) |
| 2 | 77 | M | Nivolumab + ipilimumab | Melanoma | 111 | 2 | 1 | Steroid | Hypertension | Hepatitis | Dead  (51) |
| 3 | 52 | F | Nivolumab + ipilimumab | Kidney cancer | 219 | 3 | 1 | Conservative treatment | Hyperlipidemia | Arthritis | Alive  (40) |
| 4 | 60 | M | Nivolumab + ipilimumab | Melanoma | 116 | 3 | 1 | Steroid | Diabetes mellitus | Arthritis | Alive  (26) |
| 5 | 79 | F | Nivolumab + ipilimumab | Melanoma | 54 | 1 | 1 | Steroid | - | Endocrine dysfunction | Dead  (13) |
| 6 | 72 | M | Pembrolizumab | Kidney cancer | 27 | 2 | 1 | Steroid | Hypertension | - | Dead  (4) |
| 7 | 47 | M | Nivolumab | Esophageal cancer | 249 | 3 | 1 | Conservative treatment | - | - | Alive  (31) |
| 8 | 80 | F | Pembrolizumab | NSCLC | 119 | 1 | 1 | Conservative treatment | Hypertension/  hyperlipidemia | - | Dead  (10) |
| 9 | 50 | M | Nivolumab | Melanoma | 65 | 3 | 2 | Conservative treatment | - | Rash | Alive  (19) |
| 10 | 82 | M | Pembrolizumab | NSCLC | 149 | 1 | 1 | Conservative treatment | Asthma | - | Dead  (13) |
| 11 | 65 | M | Pembrolizumab | NSCLC | 64 | 3 | 3 | Steroid/  infliximab | - | Rash | Dead  (27) |
| 12 | 65 | F | Pembrolizumab | Cancer of unknown primary | 21 | 3 | 1 | Steroid | - | Endocrine dysfunction | Alive  (39) |
| 13 | 71 | M | Nivolumab | NSCLC | 231 | 2 | 1 | Steroid | - | Rash | Alive  (51) |
| 14 | 77 | M | Nivolumab | Esophageal cancer | 147 | 3 | 3 | Steroid/  infliximab | - | - | Dead  (24) |
| 15 | 76 | M | Pembrolizumab | NSCLC | 218 | 3 | 3 | Steroid | - | Endocrine dysfunction | Alive  (30) |
| 16 | 69 | F | Nivolumab | Melanoma | 105 | 3 | 3 | Steroid | - | Endocrine dysfunction | Alive  (24) |
| 17 | 48 | M | Nivolumab + ipilimumab | NSCLC | 14 | 2 | 1 | Conservative treatment | Hypertension | Rash | Alive  (18) |
| 18 | 57 | M | Nivolumab | Malignant pleural mesothelioma | 56 | 2 | 1 | Steroid | Hypertension | - | Alive  (17) |
| 19 | 75 | M | Nivolumab | Kidney cancer | 837 | 1 | 2 | Conservative treatment | Epilepsy | - | Alive  (39) |
| 20 | 69 | F | Pembrolizumab | Kidney cancer | 594 | 2 | 2 | Steroid | Diabetes mellitus | Rash | Alive  (43) |
| 21 | 57 | M | Pembrolizumab | Kidney cancer | 47 | 3 | 1 | Steroid | Schizophrenia | - | Dead  (10) |
| 22 | 71 | M | Pembrolizumab | Bladder cancer | 60 | 2 | 1 | Steroid | - | - | Dead  (4) |
| 23 | 73 | M | Atezolizumab | NSCLC | 37 | 2 | 1 | Steroid | - | - | Alive  (41) |
| 24 | 70 | F | Durvalumab | NSCLC | 20 | 2 | 2 | Steroid | - | - | Alive  (34) |
| 25 | 80 | M | Pembrolizumab | Melanoma | 73 | 3 | 3 | Steroid/  infliximab | - | - | Dead  (8) |
| 26 | 69 | M | Nivolumab | Melanoma | 334 | 3 | 3 | Steroid/  infliximab | Hypertension/  diabetes mellitus | - | Alive  (14) |

Abbreviations: CTCAE, Common Terminology Criteria for Adverse Events; F, female; ICI, immune checkpoint inhibitor; M, male; NSCLC, non-small cell lung cancer.

***^a^***Case 1 was treated with atezolizumab together with paclitaxel, carboplatin, and bevacizumab.

**Supplementary Table S2. Characteristics of the patients with ICI-induced colitis**

| **Number of patients, n** | 26 |
| --- | --- |
| Median age, years (range) | 69.5 (47–82) |
| Sex |  |
| Male, n (%) | 18 (69.2) |
| Female, n (%) | 8 (30.8) |
|  |  |
| **Cancer type, n (%)** |  |
| Non-small-cell lung cancer | 8 (30.8) |
| Melanoma | 7 (26.9) |
| Kidney cancer | 5 (19.2) |
| Esophageal cancer | 2 (7.7) |
| Peritoneal cancer | 1 (3.8) |
| Cancer of unknown primary | 1 (3.8) |
| Malignant pleural mesothelioma | 1 (3.8) |
| Bladder cancer | 1 (3.8) |
|  |  |
| **ICI medication, n (%)** |  |
| Pembrolizumab | 10 (38.5) |
| Nivolumab | 8 (30.8) |
| Nivolumab and ipilimumab | 5 (19.2) |
| Atezolizumab | 2 (7.7) |
| Durvalumab | 1 (3.8) |
|  |  |
| **Time to ICI-induced colitis onset (days), median (range)** | 108 (14–837) |
|  |  |
| **Grade ≥3 adverse events, n (%)** |  |
| Diarrhea | 12 (46.2) |
| Colitis | 7 (26.9) |
|  |  |
| **Treatment, n (%)** |  |
| Steroid alone | 15 (57.7) |
| Conservative treatment | 7 (26.9) |
| Steroid/infliximab | 4 (15.4) |
|  |  |
| **Median follow-up time, months (range)** | 25 (4-61) |
|  |  |
| **Prognosis, n (%)** |  |
| Alive | 16 (61.5) |
| Dead | 10 (38.5) |

NOTE. Severity of diarrhea and colitis was assessed according to Common Terminology Criteria for Adverse Events (CTCAE) version 5.0 [2].

Abbreviations: ICI, immune checkpoint inhibitor.

**Supplementary Table S3. Clinical information of controls**

| **Case** | **Control** | **Age**  **(years)** | **Sex** | **Primary malignancy** | **Regimen** | **irAE category** | **CTCAE grade** |
| --- | --- | --- | --- | --- | --- | --- | --- |
| 1 | IrAE 1 | 58 | F | Breast cancer | Atezolizumab + nab-paclitaxel | Hepatitis | 4 |
| 2 | IrAE 2 | 90 | F | Cancer of unknown primary | Nivolumab | Hepatitis | 3 |
| 3 | IrAE 3 | 55 | M | Melanoma | Nivolumab + ipilimumab | Hepatitis | 3 |
| 4 | IrAE 4 | 75 | M | Melanoma | Nivolumab + ipilimumab | Hepatitis | 2 |
| 5 | IrAE 5 | 64 | M | Kidney cancer | Pembrolizumab + axitinib | Hepatitis | 3 |
| 6 | IrAE 6 | 40 | F | Melanoma | Nivolumab | Hepatitis | 3 |
| 7 | IrAE 7 | 83 | M | Malignant pleural mesothelioma | Nivolumab | Hepatitis | 3 |
| 8 | IrAE 8 | 71 | M | Kidney cancer | Nivolumab | Hepatitis | 1 |
| 9 | IrAE 9 | 70 | F | NSCLC | Atezolizumab + bevacizumab + carboplatin + paclitaxel | Hepatitis | 1 |
| 10 | IrAE 10 | 90 | M | Bladder cancer | Pembrolizumab | Hepatitis | 3 |
| 11 | IrAE 11 | 63 | M | Melanoma | Nivolumab | Hepatitis | 3 |
| 12 | IrAE 12 | 53 | M | NSCLC | Atezolizumab + carboplatin + pemetrexed | Hepatitis | 1 |
| 13 | IrAE 13 | 65 | M | SCLC | Atezolizumab + carboplatin + etoposide | Hepatitis | 2 |
| 14 | IrAE 14 | 64 | M | SCLC | Atezolizumab + carboplatin + etoposide | Hepatitis | 2 |
| 15 | IrAE 15 | 66 | M | Melanoma | Nivolumab + ipilimumab | Hepatitis | 4 |
| 16 | IrAE 16 | 72 | M | NSCLC | Pembrolizumab + carboplatin + pemetrexed | Hepatitis | 3 |
| 17 | IrAE 17 | 57 | M | Gastric cancer | Nivolumab | Hepatitis | 2 |
| 18 | IrAE 18 | 66 | F | Head and neck | Nivolumab | Hepatitis | 3 |
| 19 | IrAE 19 | 58 | M | Head and neck | Pembrolizumab | Endocrine dysfunction | 2 |
| 20 | IrAE 20 | 50 | M | Melanoma | Nivolumab + ipilimumab | Endocrine dysfunction | 2 |
| 21 | IrAE 21 | 58 | F | Melanoma | Nivolumab | Endocrine dysfunction | 2 |
| 22 | IrAE 22 | 73 | M | NSCLC | Pembrolizumab + pemetrexed | Endocrine dysfunction | 2 |
| 23 | IrAE 23 | 76 | F | NSCLC | Pembrolizumab | Endocrine dysfunction | 3 |
| 24 | IrAE 24 | 65 | M | NSCLC | Atezolizumab | Endocrine dysfunction | 1 |
| 25 | IrAE 25 | 76 | M | Gastric cancer | Nivolumab | Endocrine dysfunction | 2 |
| 26 | IrAE 26 | 62 | M | NSCLC | Pembrolizumab | Endocrine dysfunction | 2 |
| 27 | IrAE 27 | 72 | M | Esophageal cancer | Nivolumab | Endocrine dysfunction | 2 |
| 28 | IrAE 28 | 75 | M | Melanoma | Nivolumab + ipilimumab | Endocrine dysfunction | 2 |
| 29 | IrAE 29 | 85 | M | Melanoma | Nivolumab + ipilimumab | Endocrine dysfunction | 4 |
| 30 | IrAE 30 | 55 | M | Melanoma | Nivolumab + ipilimumab | Pneumonitis | 2 |
| 31 | IrAE 31 | 51 | F | NSCLC | Pembrolizumab | Pneumonitis | 3 |
| 32 | IrAE 32 | 63 | M | Melanoma | Nivolumab | Pneumonitis | 3 |
| 33 | IrAE 33 | 80 | M | NSCLC | Durvalumab | Pneumonitis | 4 |
| 34 | IrAE 34 | 67 | M | Gastric cancer | Nivolumab | Pneumonitis | 1 |
| 35 | IrAE 35 | 54 | M | Head and neck | Nivolumab | Pneumonitis | 3 |
| 36 | IrAE 36 | 55 | M | SCLC | Nivolumab | Acute kidney injury | 4 |
| 37 | IrAE 37 | 74 | M | Malignant pleural mesothelioma | Nivolumab | Myasthenia gravis | 4 |
| 38 | IrAE 38 | 78 | M | NSCLC | Atezolizumab | Myasthenia gravis | 4 |
| 39 | IrAE 39 | 49 | M | NSCLC | Nivolumab + carboplatin + pemetrexed | Hemolytic uremic syndrome | 3 |
| 40 | Cancer 1 | 57 | M | Pancreatic cancer | - | - | - |
| 41 | Cancer 2 | 73 | F | Pancreatic cancer | - | - | - |
| 42 | Cancer 3 | 70 | F | Pancreatic cancer | - | - | - |
| 43 | Cancer 4 | 63 | F | Pancreatic cancer | - | - | - |
| 44 | Cancer 5 | 77 | M | Pancreatic cancer | - | - | - |
| 45 | Cancer 6 | 83 | M | Pancreatic cancer | - | - | - |
| 46 | Cancer 7 | 64 | F | Pancreatic cancer | - | - | - |
| 47 | Cancer 8 | 42 | F | Pancreatic cancer | - | - | - |
| 48 | Cancer 9 | 74 | M | Pancreatic cancer | - | - | - |
| 49 | Cancer 10 | 74 | F | Pancreatic cancer | - | - | - |
| 50 | Cancer 11 | 72 | M | Pancreatic cancer | - | - | - |
| 51 | Cancer 12 | 61 | M | Pancreatic cancer | - | - | - |
| 52 | Cancer 13 | 79 | M | Bile duct cancer | - | - | - |
| 53 | Cancer 14 | 81 | M | Bile duct cancer | - | - | - |
| 54 | Cancer 15 | 66 | M | Bile duct cancer | - | - | - |
| 55 | Cancer 16 | 63 | F | Bile duct cancer | - | - | - |
| 56 | Cancer 17 | 60 | M | Bile duct cancer | - | - | - |
| 57 | Cancer 18 | 71 | M | Bile duct cancer | - | - | - |
| 58 | Cancer 19 | 68 | F | Bile duct cancer | - | - | - |
| 59 | Cancer 20 | 69 | F | Bile duct cancer | - | - | - |
| 60 | Cancer 21 | 71 | M | Bile duct cancer | - | - | - |
| 61 | Cancer 22 | 57 | M | Bile duct cancer | - | - | - |
| 62 | Cancer 23 | 69 | M | Bile duct cancer | - | - | - |
| 63 | Cancer 24 | 69 | M | Bile duct cancer | - | - | - |
| 64 | Cancer 25 | 85 | M | Bile duct cancer | - | - | - |
| 65 | Cancer 26 | 74 | M | Bile duct cancer | - | - | - |
| 66 | Cancer 27 | 57 | F | Bile duct cancer | - | - | - |
| 67 | Cancer 28 | 33 | M | Colon cancer | - | - | - |
| 68 | Cancer 29 | 86 | M | Colon cancer | - | - | - |
| 69 | Cancer 30 | 67 | M | Colon cancer | - | - | - |
| 70 | Cancer 31 | 49 | F | Colon cancer | - | - | - |
| 71 | Cancer 32 | 57 | M | Colon cancer | - | - | - |
| 72 | Cancer 33 | 79 | M | Colon cancer | - | - | - |
| 73 | Cancer 34 | 72 | M | Colon cancer | - | - | - |
| 74 | Cancer 35 | 85 | F | Colon cancer | - | - | - |
| 75 | Cancer 36 | 69 | M | Colon cancer | - | - | - |
| 76 | Cancer 37 | 71 | F | Colon cancer | - | - | - |
| 77 | Cancer 38 | 93 | F | Colon cancer | - | - | - |
| 78 | Cancer 39 | 74 | M | Colon cancer | - | - | - |
| 79 | Cancer 40 | 69 | F | Colon cancer | - | - | - |
| 80 | Cancer 41 | 78 | F | Colon cancer | - | - | - |
| 81 | Cancer 42 | 76 | M | Colon cancer | - | - | - |
| 82 | Cancer 43 | 46 | F | Colon cancer | - | - | - |
| 83 | Cancer 44 | 73 | F | Gastric cancer | - | - | - |
| 84 | Cancer 45 | 69 | M | Gastric cancer | - | - | - |
| 85 | Cancer 46 | 59 | F | Gastric cancer | - | - | - |
| 86 | Cancer 47 | 69 | M | Gastric cancer | - | - | - |
| 87 | Cancer 48 | 79 | M | Gastric cancer | - | - | - |
| 88 | Cancer 49 | 67 | M | Gastric cancer | - | - | - |
| 89 | Cancer 50 | 58 | M | Gastric cancer | - | - | - |
| 90 | Cancer 51 | 71 | M | Gastric cancer | - | - | - |
| 91 | Cancer 52 | 55 | M | Gastric cancer | - | - | - |
| 92 | Cancer 53 | 64 | M | Gastric cancer | - | - | - |
| 93 | Cancer 54 | 60 | M | Gastric cancer | - | - | - |
| 94 | Cancer 55 | 63 | F | Gastric cancer | - | - | - |
| 95 | Cancer 56 | 54 | F | Gastric cancer | - | - | - |
| 96 | Cancer 57 | 74 | M | Gastric cancer | - | - | - |
| 97 | Cancer 58 | 68 | F | Gastric cancer | - | - | - |
| 98 | Cancer 59 | 40 | M | NSCLC | - | - | - |
| 99 | Cancer 60 | 52 | M | NSCLC | - | - | - |
| 100 | Cancer 61 | 54 | F | NSCLC | - | - | - |
| 101 | Cancer 62 | 72 | M | NSCLC | - | - | - |
| 102 | Cancer 63 | 76 | M | NSCLC | - | - | - |
| 103 | Cancer 64 | 79 | M | NSCLC | - | - | - |
| 104 | Cancer 65 | 74 | M | NSCLC | - | - | - |
| 105 | Cancer 66 | 71 | M | NSCLC | - | - | - |
| 106 | Cancer 67 | 49 | M | NSCLC | - | - | - |
| 107 | Cancer 68 | 69 | F | NSCLC | - | - | - |
| 108 | Cancer 69 | 38 | M | NSCLC | - | - | - |
| 109 | Cancer 70 | 82 | F | NSCLC | - | - | - |
| 110 | Cancer 71 | 70 | F | NSCLC | - | - | - |
| 111 | Cancer 72 | 49 | M | NSCLC | - | - | - |
| 112 | Cancer 73 | 62 | M | NSCLC | - | - | - |
| 113 | Cancer 74 | 62 | M | Melanoma | - | - | - |
| 114 | Cancer 75 | 92 | F | Melanoma | - | - | - |
| 115 | Cancer 76 | 72 | F | Melanoma | - | - | - |
| 116 | Cancer 77 | 81 | M | Melanoma | - | - | - |
| 117 | HV1 | 33 | M | - | - | - | - |
| 118 | HV2 | 35 | M | - | - | - | - |
| 119 | HV3 | 35 | F | - | - | - | - |
| 120 | HV4 | 35 | M | - | - | - | - |
| 121 | HV5 | 34 | M | - | - | - | - |
| 122 | HV6 | 33 | M | - | - | - | - |
| 123 | HV7 | 42 | M | - | - | - | - |
| 124 | HV8 | 36 | M | - | - | - | - |
| 125 | HV9 | 34 | M | - | - | - | - |
| 126 | HV10 | 35 | M | - | - | - | - |
| 127 | HV11 | 32 | F | - | - | - | - |
| 128 | HV12 | 21 | M | - | - | - | - |
| 129 | HV13 | 52 | M | - | - | - | - |
| 130 | HV14 | 34 | M | - | - | - | - |
| 131 | HV15 | 27 | F | - | - | - | - |
| 132 | HV16 | 37 | M | - | - | - | - |
| 133 | HV17 | 33 | M | - | - | - | - |
| 134 | HV18 | 32 | M | - | - | - | - |
| 135 | HV19 | 33 | F | - | - | - | - |
| 136 | HV20 | 25 | M | - | - | - | - |
| 137 | HV21 | 29 | F | - | - | - | - |
| 138 | HV22 | 36 | M | - | - | - | - |
| 139 | HV23 | 33 | M | - | - | - | - |
| 140 | HV24 | 24 | F | - | - | - | - |
| 141 | HV25 | 37 | M | - | - | - | - |
| 142 | HV26 | 42 | M | - | - | - | - |
| 143 | HV27 | 38 | M | - | - | - | - |
| 144 | HV28 | 71 | F | - | - | - | - |
| 145 | HV29 | 83 | M | - | - | - | - |
| 146 | HV30 | 77 | M | - | - | - | - |
| 147 | HV31 | 83 | M | - | - | - | - |
| 148 | HV32 | 66 | M | - | - | - | - |
| 149 | HV33 | 67 | M | - | - | - | - |
| 150 | HV34 | 66 | F | - | - | - | - |
| 151 | HV35 | 38 | M | - | - | - | - |
| 152 | HV36 | 36 | M | - | - | - | - |
| 153 | HV37 | 33 | M | - | - | - | - |
| 154 | HV38 | 35 | M | - | - | - | - |
| 155 | HV39 | 33 | F | - | - | - | - |
| 156 | HV40 | 42 | F | - | - | - | - |
| 157 | HV41 | 33 | M | - | - | - | - |

NOTE. All cancer patients were diagnosed by histology, and irAEs were diagnosed according to the ASCO or ESMO clinical practice guidelines [3, 4]. Sera of six healthy volunteers (HV2, HV3, HV6, HV13, HV24, and HV30) were used to examine subclasses and isotypes of anti-integrin αvβ6 autoantibodies (Supplementary Figure S1) and for measurement of the autoantibodies against other integrins (Supplementary Figure S3). Sera of 12 healthy volunteers (HV1, HV2, HV6, HV8, HV11, HV13, HV16, HV24, HV25, HV26, HV27, and HV30) were used to purify IgG to analyze the inhibitory activity against integrin αvβ6-fibronectin binding (Figure 4).

Abbreviations: ASCO, American Society of Clinical Oncology; CTCAE, Common Terminology Criteria for Adverse Events; ESMO, European Society for Medical Oncology; F, female; HV, healthy volunteer; irAE, immune-related adverse events; M, male; NSCLC, non-small cell lung cancer; SCLC, small cell lung cancer.

**Supplementary Table S4. Clinical information of patients with UC**

| **Sample** | **Age**  **(years)** | **Sex** | **CRP*^a^* (mg/dL)** | **Extent of disease** | **Mayo score***^b^* | | **Treatment** | **Used for the data in Supplementary Figure S3** | **Used for the data in Figure 4** |
| --- | --- | --- | --- | --- | --- | --- | --- | --- | --- |
|  |  |  |  |  | **Full** | **Partial** |  |  |  |
| UC 1 | 58 | F | 0.9 | Left-sided colitis | 8 | 6 | Mesalazine, mesalazine suppository, azathioprine | + | + |
| UC 2 | 50 | M | 1.3 | Pancolitis | 5 | 2 | Mesalazine, mesalazine suppository | - | + |
| UC 3 | 45 | F | <0.1 | Pancolitis | 0 | 0 | Salazosulfapyridine | + | + |
| UC 4 | 48 | F | <0.1 | Pancolitis | 1 | 0 | Mesalazine, prednisolone, tacrolimus | + | + |
| UC 5 | 33 | M | <0.1 | Pancolitis | 0 | 0 | Mesalazine | - | + |
| UC 6 | 30 | M | <0.1 | Pancolitis | 3 | 2 | Mesalazine | - | + |
| UC 7 | 43 | F | 2.6 | Pancolitis | 3 | 1 | No medication | + | + |
| UC 8 | 44 | M | 0.3 | Left-sided colitis | 7 | 4 | Mesalazine | + | + |
| UC 9 | 35 | F | <0.1 | Pancolitis | 8 | 5 | Salazosulfapyridine, mesalazine suppository, 6MP, vedolizumab | - | + |
| UC 10 | 24 | F | <0.1 | Pancolitis | 2 | 0 | Mesalazine, azathioprine | - | + |
| UC 11 | 35 | M | <0.1 | Left-sided colitis | 0 | 0 | Mesalazine | + | + |
| UC 12 | 29 | M | 0.3 | Pancolitis | 8 | 5 | Mesalazine, prednisolone, azathioprine | - | + |

*^a^*The normal range of CRP is 0-0.2 mg/dL

*^b^*Reference 5

Abbreviations: CRP, C-reactive protein; F, female; M, male; 6MP, mercaptopurine; UC, ulcerative colitis.

**Supplementary Table S5. Categorization of endoscopic findings of typical UC**

| **Typical UC findings***^a^* |
| --- |
| 1. Diffuse and continuous erythema |
| 2. Continuous disease from the rectum |
| 3. Granularity |
| 4. Loss of vascular pattern |
| 5. Bleeding |
| 6. Ulceration |

*^a^*Reference 6

Abbreviations: UC, ulcerative colitis.

**Supplementary Table S6. Endoscopic scores in patients with ICI-induced colitis evaluated by two experienced endoscopists**

|  | **Finding by Reviewer 1** | **Case 1** | **Case 2** | **Case 3** | **Case 4** | **Case 5** | **Case 6** | **Case 7** | **Case 8** | **Case 9** | **Case 10** | **Case 11** | **Case 12** | **Case 13** | **Case 14** | **Case 15** | **Case 16** | **Case 17** | **Case 18** | **Case 19** | **Case 20** | **Case 21** | **Case 22** | **Case 23** | **Case 24** | **Case 25** | **Case 26** |
| --- | --- | --- | --- | --- | --- | --- | --- | --- | --- | --- | --- | --- | --- | --- | --- | --- | --- | --- | --- | --- | --- | --- | --- | --- | --- | --- | --- |
| **1** | **Diffuse and continuous erythema** | 1 | 0 | 0 | 1 | 1 | 0 | 0 | 0 | 0 | 1 | 1 | 0 | 0 | 1 | 0 | 1 | 0 | 0 | 1 | 1 | 0 | 0 | 0 | 0 | 1 | 1 |
| **2** | **Continuous disease from rectum** | 1 | 0 | 0 | 1 | 1 | 0 | 0 | 0 | 0 | 1 | 1 | 0 | 0 | 1 | 0 | 1 | 0 | 1 | 1 | 1 | 0 | 1 | 0 | 0 | 1 | 1 |
| **3** | **Granularity** | 1 | 1 | 0 | 0 | 1 | 0 | 0 | 1 | 0 | 0 | 1 | 0 | 1 | 1 | 0 | 1 | 0 | 1 | 1 | 1 | 0 | 0 | 0 | 0 | 1 | 1 |
| **4** | **Loss of vascular pattern** | 1 | 1 | 0 | 1 | 1 | 1 | 1 | 1 | 0 | 1 | 1 | 0 | 0 | 1 | 1 | 1 | 0 | 1 | 1 | 1 | 1 | 1 | 0 | 0 | 1 | 1 |
| **5** | **Bleeding** | 0 | 0 | 0 | 0 | 0 | 0 | 0 | 0 | 0 | 0 | 0 | 0 | 0 | 1 | 0 | 0 | 0 | 0 | 0 | 0 | 0 | 0 | 0 | 0 | 0 | 0 |
| **6** | **Ulceration** | 0 | 1 | 0 | 0 | 0 | 0 | 0 | 0 | 0 | 0 | 0 | 0 | 0 | 0 | 0 | 0 | 0 | 0 | 1 | 0 | 0 | 0 | 0 | 0 | 1 | 0 |
|  | **Total** | **4** | **3** | **0** | **3** | **4** | **1** | **1** | **2** | **0** | **3** | **4** | **0** | **1** | **5** | **1** | **4** | **0** | **3** | **5** | **4** | **1** | **2** | **0** | **0** | **5** | **4** |

|  | **Finding by Reviewer 2** | **Case 1** | **Case 2** | **Case 3** | **Case 4** | **Case 5** | **Case 6** | **Case 7** | **Case 8** | **Case 9** | **Case 10** | **Case 11** | **Case 12** | **Case 13** | **Case 14** | **Case 15** | **Case 16** | **Case 17** | **Case 18** | **Case 19** | **Case 20** | **Case 21** | **Case 22** | **Case 23** | **Case 24** | **Case 25** | **Case 26** |
| --- | --- | --- | --- | --- | --- | --- | --- | --- | --- | --- | --- | --- | --- | --- | --- | --- | --- | --- | --- | --- | --- | --- | --- | --- | --- | --- | --- |
| **1** | **Diffuse and continuous erythema** | 1 | 1 | 0 | 1 | 1 | 0 | 0 | 1 | 0 | 1 | 1 | 0 | 0 | 1 | 0 | 1 | 0 | 0 | 1 | 1 | 0 | 1 | 0 | 0 | 1 | 1 |
| **2** | **Continuous disease from rectum** | 1 | 0 | 0 | 1 | 1 | 1 | 1 | 0 | 0 | 0 | 1 | 0 | 0 | 1 | 1 | 1 | 0 | 0 | 1 | 1 | 0 | 1 | 0 | 0 | 1 | 1 |
| **3** | **Granularity** | 1 | 1 | 0 | 1 | 1 | 0 | 0 | 1 | 0 | 1 | 1 | 0 | 1 | 1 | 0 | 1 | 0 | 0 | 1 | 1 | 0 | 0 | 0 | 0 | 1 | 1 |
| **4** | **Loss of vascular pattern** | 1 | 1 | 1 | 1 | 1 | 1 | 1 | 1 | 1 | 1 | 1 | 1 | 1 | 1 | 1 | 1 | 1 | 0 | 1 | 1 | 1 | 1 | 0 | 1 | 1 | 1 |
| **5** | **Bleeding** | 0 | 0 | 0 | 0 | 0 | 0 | 0 | 0 | 0 | 0 | 0 | 0 | 0 | 1 | 0 | 1 | 0 | 0 | 1 | 0 | 0 | 0 | 0 | 0 | 1 | 1 |
| **6** | **Ulceration** | 1 | 1 | 0 | 0 | 0 | 0 | 0 | 0 | 0 | 1 | 0 | 0 | 0 | 0 | 0 | 0 | 0 | 0 | 1 | 0 | 0 | 0 | 0 | 0 | 0 | 0 |
|  | **Total** | **5** | **4** | **1** | **4** | **4** | **2** | **2** | **3** | **1** | **4** | **4** | **1** | **2** | **5** | **2** | **5** | **1** | **0** | **6** | **4** | **1** | **3** | **0** | **1** | **5** | **5** |

NOTE. The patient's endoscopic findings were categorized into typical UC findings, as shown in Supplementary Table S5, and each finding was calculated as one point.

Abbreviations: ICI, immune checkpoint inhibitor; UC, ulcerative colitis.

**Supplementary Table S7. Antigens used for enzyme-linked immunosorbent assays**

| **Proteins** | **Product code** | **Company** | **State** | **Country** |
| --- | --- | --- | --- | --- |
| Recombinant Human Integrin αvβ1 | IT1-H52E1-100ug | ACROBiosystems | Delaware | United States |
| Recombinant Human Integrin αvβ3 | IT3-H52E3-100ug | ACROBiosystems | Delaware | United States |
| Recombinant Human Integrin αvβ5 | IT5-H52W5-100ug | ACROBiosystems | Delaware | United States |
| Recombinant Human Integrin αvβ6 | IT6-H52E1-100ug | ACROBiosystems | Delaware | United States |
| Recombinant Human Integrin αvβ8 | IT8-H52W4-100ug | ACROBiosystems | Delaware | United States |
| Recombinant Human Integrin β6 | - | Medical and Biological Laboratories | Tokyo | Japan |
| Recombinant Human Integrin α8β1 | IT1-H52W9-100ug | ACROBiosystems | Delaware | United States |

**Supplementary References**

1. Kuwada T, Shiokawa M, Kodama Y, Ota S, Kakiuchi N, Nannya Y, et al. Identification of an Anti–Integrin αvβ6 Autoantibody in Patients With Ulcerative Colitis. Gastroenterology 2021;160:2383-94.e21.
2. Common Terminology Criteria for Adverse Events (CTCAE) v5.0. https://ctep.cancer.gov/protocoldevelopment/electronic_applications/docs/CTCAE_v5_ Quick_Reference_8.5x11.pdf 2018.
3. Schneider BJ, Naidoo J, Santomasso BD, Lacchetti C, Adkins S, Anadkat M, et al. Management of Immune-Related Adverse Events in Patients Treated With Immune Checkpoint Inhibitor Therapy: ASCO Guideline Update. J Clin Oncol 2021;39:4073-126.
4. Haanen J, Obeid M, Spain L, Carbonnel F, Wang Y, Robert C, et al. Management of toxicities from immunotherapy: ESMO Clinical Practice Guideline for diagnosis, treatment and follow-up. Ann Oncol 2022;33:1217-38.
5. Schroeder KW, Tremaine WJ, Ilstrup DM. Coated oral 5-aminosalicylic acid therapy for mildly to moderately active ulcerative colitis. A randomized study. N Engl J Med 1987;317:1625-9.
6. Magro F, Gionchetti P, Eliakim R, Ardizzone S, Armuzzi A, Barreiro-de Acosta M, et al. Third European Evidence-based Consensus on Diagnosis and Management of Ulcerative Colitis. Part 1: Definitions, Diagnosis, Extra-intestinal Manifestations, Pregnancy, Cancer Surveillance, Surgery, and Ileo-anal Pouch Disorders. J Crohns Colitis 2017;11:649-70.
